# Membrane Lipid Remodeling Strategies Regulate Fluidity for Acute Temperature Adaptation in Oysters

**DOI:** 10.1111/eva.70156

**Published:** 2025-09-13

**Authors:** Mingyang Du, Jincheng Chen, Chaogang Wang, Zhuxiang Jiang, Min Wang, Meiqian Pang, Tian Bu, Rihao Cong, Wei Wang, Guofan Zhang, Li Li

**Affiliations:** ^1^ Key Laboratory of Breeding Biotechnology and Sustainable Aquaculture (CAS) Institute of Oceanology, Chinese Academy of Sciences Qingdao China; ^2^ Laboratory for Marine Biology and Biotechnology, Qingdao Marine Science and Technology Center Qingdao China; ^3^ Laboratory of Experimental Marine Biology Institute of Oceanology, Chinese Academy of Sciences Qingdao China; ^4^ University of Chinese Academy of Sciences Beijing China; ^5^ Laboratory for Marine Fisheries Science and Food Production Processes, Qingdao Marine Science and Technology Center Qingdao China; ^6^ National and Local Joint Engineering Laboratory of Ecological Mariculture Qingdao China; ^7^ Shandong Center of Technology Innovation for Oyster Seed Industry Qingdao China; ^8^ Southern Marine Science and Engineering Guangdong Laboratory (Zhanjiang) Zhanjiang China

**Keywords:** global warming, homeoviscous adaptation, lipid remodeling, oysters, plasma membrane

## Abstract

Extreme climatic temperature stress induced by global warming poses a severe threat to the survival of marine invertebrates. The plasma membrane functions as a natural barrier and serves as the first responder to ambient temperature through dynamic modulation of its fluidity. However, the adaptive mechanisms of membrane lipid remodeling in response to temperature fluctuations remain poorly understood in marine organisms. Oysters, the most widely cultivated shellfish globally, hold significant economic and ecological importance. We characterized the changes in plasma membrane lipid composition of two congeneric oyster species—the northern/cold‐adapted *Crassostrea gigas* and the southern/warm‐adapted *Crassostrea angulata* —under short‐term acute heat and cold stress, including changes in lipid subclass content, glycerophospholipid acyl chain length, and glycerophospholipid unsaturation. Our results revealed sphingolipids and sterol lipids content may play a more critical role in short‐term temperature adaptation, while glycerophospholipid alterations may prioritize dynamic lipid modifications over abundance changes. Notably, the relatively cold tolerant *C. gigas* exhibited higher lipid unsaturation and shorter acyl chain lengths, with a preferential modulation of glycerophospholipid acyl chain length, while the heat tolerant *C. angulata* regulated fatty acid unsaturation to maintain membrane fluidity for temperature adaptation. Divergent membrane lipid remodeling strategies in two congeneric oysters provide new insights into the adaptation mechanisms of membrane fluidity in marine organisms, informing risk assessment for aquaculture industries under global warming. The identification of key components such as phosphatidylethanolamine, sphingosine, ceramide phosphates, and cold and heat adapted lipid molecules provides important biomarkers for predicting the adaptive potential of marine organisms to future extreme climate.

## 1 Introduction

The rise in seawater temperature induced by global warming, accompanied with the increasing occurrence of extreme climatic events such as marine heatwaves, poses a critical survival threat to marine organisms (Monteiro et al. 2023; Starko et al. 2024; Witman et al. 2023). Since the 1950s, sea surface temperatures (SST) have been consistently increasing, with a rise of 0.11°C (0.19°F) by the year 2020 (Hutchins and Tagliabue 2024; Venegas et al. 2023). Aside from the continuous increase in ocean temperatures, extreme events pose short‐term acute temperature stress to organisms, which is currently a widely concerned and prevalent phenomenon (Harris et al. 2018; Smith et al. 2023). The ambient temperature is an important factor in the growth and development of organisms, as its fluctuations affect the rigidity of biological membranes (Ernst et al. 2016), enzyme activity (Wang and Swartz 2024), immune function (Karl et al. 2011), and reproductive capacity (Munns and Millar 2023), among other important physiological processes. Therefore, given the intensified temperature changes in the marine environment, there is an urgent need to explore key targets for acute temperature stress and study the adaptation strategies of marine organisms to improve their adaptive capacity.

The plasma membrane acts as a natural barrier for cells and is sensitive to environmental stresses, especially temperature changes that impact membrane fluidity (Ernst et al. 2016), permeability (Niu and Xiang 2018) and cellular toxicity (Vígh et al. 2007). At organism's adapted temperatures, lipids in the cell membrane exist in a liquid crystalline phase, sustaining crucial cellular functions such as regulating the passage of metabolites (Raj et al. 2023), receiving external signals for signal transduction (Niu and Xiang 2018), and maintaining stable osmotic pressure across the cell membrane (Roffay et al. 2021). As temperature increases, the movement of lipid molecules escalates, and the fatty acid tails in the phospholipid bilayer become more loosely packed, leading the membrane to become “superfluid” and lose its regular functionality (Hazel 1995). During periods of temperature decrease, the membrane bilayer transitions from a disordered fluid state to a more ordered gel phase, resulting in a reduction in membrane fluidity (Wu et al. 2023). Therefore, fluctuations in ambient temperature sensitively affect the physical properties of the membrane, thereby compromising cellular homeostasis.

To maintain membrane fluidity and functionality, poikilothermic animals, which cannot regulate their internal temperature, adjust the composition of membrane lipids dynamically through a process known as homeoviscous adaptation (Ernst et al. 2016; Renne and Ernst 2023; Wu et al. 2023). This adaptation mechanism employs organism‐specific responses to maintain the normal functional state of membranes and counteract the effects of temperature stress, involving three types of changes in lipids. Firstly, changes in the relative proportions of membrane lipids, such as phospholipids, sphingolipids, and sterols (Erimban and Daschakraborty 2020). Phospholipids are the primary constituents of cell membrane lipids and can be classified into various types like phosphatidylethanolamine (PE) and phosphatidylcholine (PC), based on the different headgroup compositions (Morita and Ikeda 2022). The polarity, size, and charge of the headgroup directly impact the physical properties and fluidity of the membrane (Chintalapati et al. 2004; Fajardo et al. 2011). Additionally, the other two major lipid components of membrane lipids, sphingolipids and sterols, are also considered crucial for maintaining membrane fluidity under environmental temperature stress (Tanwar et al. 2024; Zhu et al. 2023). Secondly, changes in the length of acyl chains of membrane lipids, especially phospholipids, which represent the predominant lipid species (Strahl and Errington 2017). Longer acyl chains increase the intermolecular forces between phospholipid molecules, causing them to pack more tightly together, resulting in an increase in membrane rigidity (Hazel 1995; Xiong et al. 2024). Finally, changes in the degree of acyl chain unsaturation. Double bonds can induce curvature in the normally straight acyl chains of phospholipids, leading to a looser packing of lipid molecules and consequently enhancing membrane fluidity (Strahl and Errington 2017; Wu et al. 2023). However, current research on adaptation mechanisms of lipid remodeling in cell membrane is predominantly focused on bacteria (Strahl and Errington 2017), nematodes (Sedensky et al. 2004), and plants (Kania et al. 2023), with limited observations in marine organisms.

Sessile oysters inhabiting the intertidal zone experience significant diurnal temperature fluctuations driven by tidal dynamics, making them sentinel organisms for sensing marine temperature change (Judge et al. 2018; Zhang et al. 2012). Oysters are the most widely cultured shellfish worldwide, possessing significant economic value, while oyster reefs, as critical marine ecosystems, provide essential ecological benefits (McLeod et al. 2020; Zhang et al. 2012). Therefore, oysters, with high phenotypic plasticity, are ideal subjects for studying the impact of severe ocean temperature fluctuations on marine organisms (Du et al. 2025; Wang et al. 2023). *Crassostrea gigas* and *Crassostrea angulata* naturally occur in the northern and southern coasts of China, respectively, shaping the divergent temperature tolerance between these two allopatric congeneric oyster species (Ren et al. 2010; Wang, Jiang, et al. 2024). *Crassostrea angulata*, native to the warmer southern regions, shows higher sublethal temperature limits and metabolic rates compared to *C. gigas* , which serves as an excellent model for studying temperature adaptation strategies in marine organisms (Ghaffari et al. 2019; Wang, Du, et al. 2024). Previous studies have observed differences in lipid content and lipid metabolism capabilities between the two closely related oyster species, majorly in the content of triglycerides, crude fat, and free fatty acids (Du et al. 2025; Li et al. 2021; Wang et al. 2021). Additionally, *C. gigas* shows significantly higher overall content of unsaturated fatty acids and desaturation index such as C18:1 oleic acid compared to *C. angulata* , potentially enhancing membrane fluidity to aid in adapting to lower temperatures (Wang et al. 2023). However, the assessment of lipid composition in the plasma membrane at a fine scale and the evaluation of adaptation strategies of membrane lipid in oysters remain poorly understood.

In this study, the indoor short‐term heat and cold stress experiments were conducted to simulate the impact of extreme marine climates on oysters and evaluate the changes in lipid composition of the plasma membrane in *C. gigas* and *C. angulata* under extreme conditions through lipid subclasses content, glycerophospholipids acyl chain length, and unsaturation levels. We revealed that the two oyster species evolved divergent adaptation strategies in response to acute temperature change through plasma membrane lipid remodeling for the first time. This suggests the existence of species‐specific tendencies in membrane lipid‐mediated adaptive strategies to temperature fluctuations among marine organisms. The identification of key lipid molecules in the plasma membrane can serve as biomarkers for assessing the adaptive capacity of membrane fluidity to temperature changes in marine organisms. Our findings provided new insights into predicting the adaptive potential of marine organisms and offered a theoretical basis for risk assessment and mitigation strategies in aquaculture industries under global warming.

## 2 Materials and Methods

### 2.1 Experimental Animals

Wild adult oysters of *C. gigas* and *C. angulata* were collected from intertidal zones in the northern (Qingdao, 35°44′N) and southern (Xiamen, 24°33′N) regions of China, respectively, from their natural habitats. The average sea surface temperature (SST) in the southern habitat was significantly higher than that in the northern habitat by 6.7°C during the past 5 years (Li et al. 2017; Li et al. 2021; Wang, Jiang, et al. 2024). In order to mitigate the impact of environmental conditions in their natural habitats, particularly temperature, on subsequent short‐term temperature stress experiments of two oyster species, we conducted a one‐generation common garden experiment. The details of the spawning experiment were based on our prior research (Du et al. 2024; Li et al. 2021). Briefly, 30 mature males and 30 females from each species were utilized to generate the F_1_ populations. The eggs obtained from 30 females were mixed and subsequently distributed into 30 separate beakers, with each beaker being fertilized with sperm from one of the 30 males. The fertilized eggs were cultured in a 100 L container at temperatures ranging from 20°C to 22°C in Laizhou City (37°18′N, Shandong Province, China). The larvae were then transferred to a 20 m^3^ outdoor pond with temperatures maintained between 18°C and 20°C. Subsequently, the F_1_ populations, aged 4 months, were transported to the sea of Muping city (37°39′N, Shandong Province, China) for further growth. Six months later, 60 *C. gigas* and 60 *C. angulata* 1‐year‐old F_1_ populations were collected and moved to Qingdao for further experiments.

### 2.2 Experimental Design: Short‐Term Acute Temperature Stress

**FIGURE 1:**
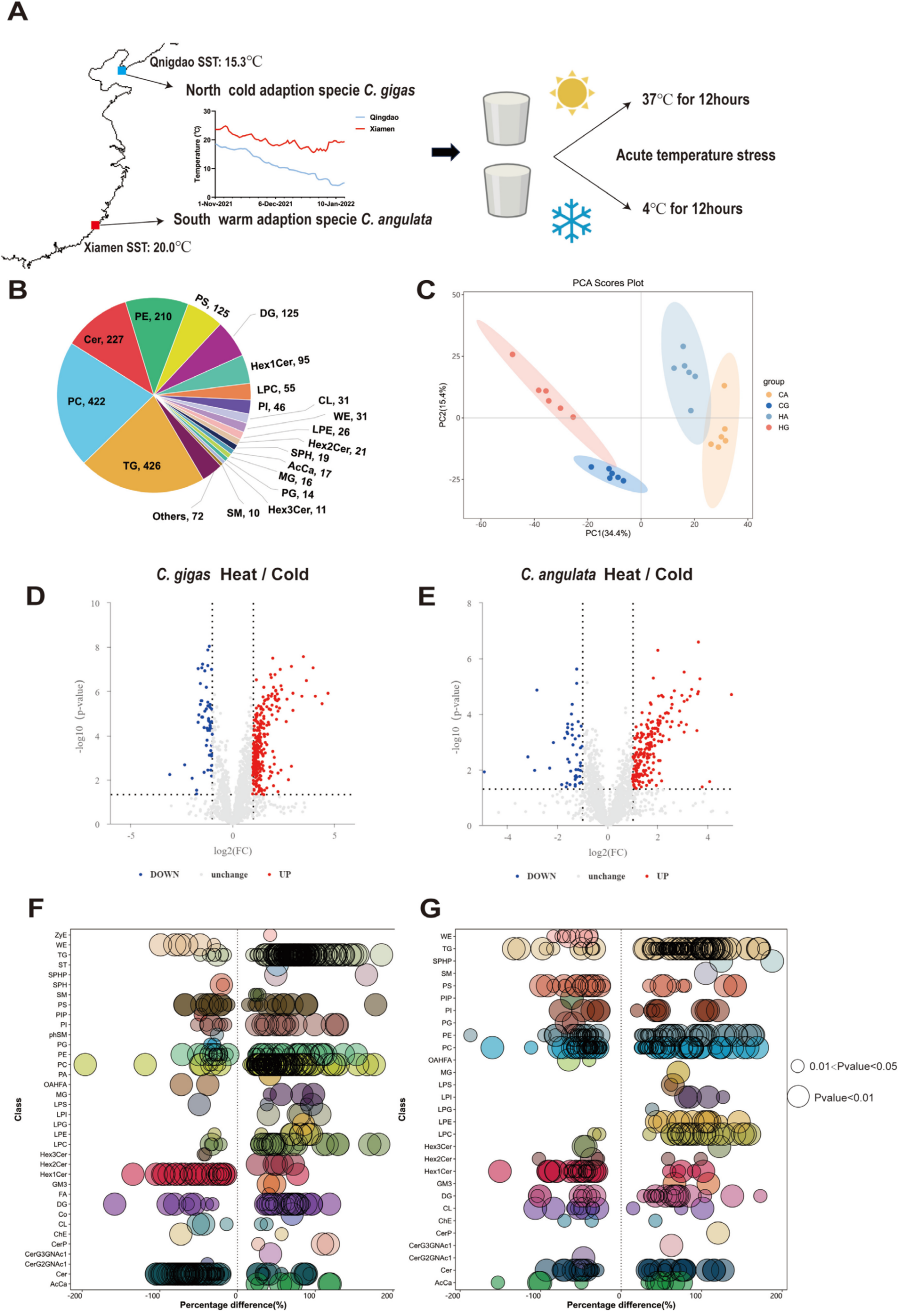
Changes in the overall lipid distribution of plasma membrane in *Crassostrea gigas* and *Crassostrea angulata* in response to short‐term acute temperature stress. (A) The details of short‐term acute temperature stress experiment design. (B) Distribution of lipid classes in all of treatment groups detected by LC–MS/MS. (C) Principal component analysis (PCA) of four treatment groups (HG, HA, CG, CA) with six biological repetitions (*n* = 6). (D, E) Log_2_ fold change of lipid molecules between heat stress versus cold stress treated in *C. gigas* (D) and *C. angulata* (E) and the corresponding significance values displayed as −log_10_(*p* value). The transverse and vertical dotted lines indicated the cutoff value for differential expression (*p* < 0.05 and fold changes > 1.5 or < 0.67). (F, G) Log_2_ fold change in lipid subclasses between heat stress versus cold stress treated in *C. gigas* (F) and *C. angulata* (G) the corresponding significance values displayed as −log_10_(*p* value). Each dot represented a lipid subclasses, and the dot size indicated significance. Smaller bubbles indicated significant differences (0.01 < *p* value < 0.05), while larger bubbles represented highly significant differences (*p* < 0.01, *n* = 6).

The congeneric oyster species were cleaned and reared in a 500 L tank with sand‐filtered and aerated seawater for 1 week to acclimate them to the survival environment and temperature. During the rearing period, the oysters were fed *Spirulina* powder, and the seawater was changed daily (water temperature 18 ± °C). Sixty *C. gigas* and sixty *C. angulata* were divided into two groups for short‐term heat and cold stress experiments, respectively. The temperature of the seawater for the heat stress treatment group was maintained at 37°C (sublethal temperature) (Ghaffari et al. 2019), while the cold stress treatment group had a seawater temperature of 4°C for a duration of 12 h; both controlled by a temperature control system (Wang et al. 2023). Before starting the acute temperature stress experiment, the water temperature was adjusted to the predetermined levels and then the oysters were placed into the water. After acute temperature stress, the gill tissues of 30 *C. gigas* and 30 *C. angulata* under heat stress (HG, HA) and 30 *C. gigas* and 30 *C. angulata* (CG, CA) under cold stress were collected and stored in a −80°C refrigerator. The detail of the experiment design is shown in Figure 1A.

### 2.3 Extraction of Plasma Membrane From Oyster Gill Tissue

The four treatment groups (HG, HA, CG, CA) with six biological replicates eachwhich were prepared by mixing gill tissues from five oysters in equal amounts. To isolate the plasma membrane, the MinuteTM Plasma Membrane Protein Isolation and Cell Fractionation Kit (SM‐005) (Invent Biotechnologies) was used. The tissue (30 mg) was ground in 500 μL of Buffer A, mixed thoroughly, and incubated uncovered on ice for 5 min. The suspension was centrifuged at 16,000 × *g* and 4°C for 30 s, and to enhance yield, the resulting pellet was resuspended and centrifuged again through the column. To isolate nuclei from membrane and cytosolic fractions, the resuspended pellet was centrifuged at 700 × *g* and 4°C for 1 min. The resulting supernatant was then transferred to a fresh tube and subjected to centrifugation at 16,000 × *g* and 4°C for 30 min. After centrifugation, the pellet containing the total membrane components was resuspended in 200 μL of Buffer B and vortexed to mix thoroughly. To separate contamination of organelle membranes in the plasma membrane fraction, the samples were centrifuged at 7800 × *g* and 4°C for 5 min. The supernatant was transferred to a new 2.0 mL reaction tube, mixed with 1.6 mL of cold PBS by inversion, and centrifuged at 16,000 × *g* for 30 min. The pellet containing the plasma membrane was collected and stored at −80°C for subsequent lipid analysis.

### 2.4 Lipid Sample Preparation and Lipidomic Assay

The extraction and mass spectrometry identification analysis of cytoplasmic membrane lipids was completed by Applied Protein Technology (Shanghai, China). Lipids were extracted using the Methyl tert‐butyl ether (MTBE) method. Briefly, after mixing the sample with water, cold methanol and MTBE were added. The mixture was then subjected to 20 min of ultrasonication at 4°C followed by a 30‐min room temperature incubation. The resulting solution was centrifuged at 10°C and 14,000 × *g* for 5 min. Equal amounts of samples from each group were mixed to create Quality Control (QC) samples, which were interspersed throughout the testing of the samples to evaluate the overall system stability during the entire experimental process. The samples were separated using the UHPLC Nexera LC‐30A ultra‐high performance liquid chromatography system (SHIMADZU). A C18 chromatographic column was employed at a column temperature of 45°C with a flow rate of 300 μL/min. The mobile phase composition was as follows: Mobile Phase A consisted of an acetonitrile‐water solution (acetonitrile: water = 6:4, v/v) with 0.1% formic acid and 0.1 μM ammonium formate, while Mobile Phase B comprised an acetonitrile‐isopropanol solution (acetonitrile: isopropanol = 1:9, v/v) with 0.1% formic acid and 0.1 μM ammonium formate.

LC–MS/MS analysis was performed on a Q Exactive plus mass spectrometer (Thermo Scientific). The detection was performed using electrospray ionization (ESI) in both positive and negative ion modes. After each full scan, 10 fragment spectra (MS^2^ scan, HCD) were collected. The resolution for MS^1^ was 70,000 at m/z 200, while the resolution for MS^2^ was 17,500 at m/z 200. LipidSearch software (Thermo Scientific) was utilized for peak identification, peak extraction, lipid identification (secondary identification), and other processing steps for lipid molecules and internal standard lipid molecules.

### 2.5 Calculation of Double Bond Index and Mean Acyl Chain Length

Double bond index (DBI) was calculated as (Vornanen et al. 1999): ∑(number of double bonds in fatty acid) × (abundance(mol%))/∑abundance(mol%) of all fatty acids in culture sample. The DBI was positively correlated with membrane fluidity.

Mean chain length (MCL) was calculated as (Vornanen et al. 1999): ∑(number of hydrocarbon chain length of fatty acid) × (abundance(mol%))/∑abundance(mol%) of all fatty acids in culture sample. The MCL was negatively correlated with membrane fluidity.

### 2.6 Statistical Analyses

All statistical analyses concerning the lipidomic data discussed in this study were derived from relative abundance measurements. The intergroup comparisons were conducted using unpaired two‐tailed Student's *t*‐tests or one‐way analysis of variance (ANOVA). Significant differences between groups were marked with “*” for *p* < 0.05, “**” for *p* < 0.01, “***” for *p* < 0.001, “****” for *p* < 0.0001. The schematic presentation was created using BioRender software (https://biorender.com).

## 3 Results

### 3.1 Acute Temperature Stress Altered the Overall Lipid Composition of Plasma Membranes in *C. gigas* and *C. angulata*

To demonstrate the reliability of data quality, we compared the Base Peak Chromatograms (BPC) of the QC samples, created a correlation plot of the QC samples, and calculated the relative standard deviations (RSD) of the QC samples. The BPC of the QC samples in positive and negative ion mode exhibits strong overlap in peak intensity and retention time (Figure S1A,B), with correlation coefficients consistently above 0.9 (Figure S1C), indicating good experimental reproducibility. The proportion of peaks with an RSD ≤ 30% exceeding 80% of the total number of peaks in the QC samples indicates good stability of the instrumental analysis system (Figure S1D). The three quality control measures ensured the reliability of the lipidomic data, making it suitable for subsequent analysis.

The lipidomic analysis identified a total of 40 lipid classes and 1999 individual lipid molecules, including 426 triglycerides (TG), 422 phosphatidylcholines (PC), 227 ceramides (Cer), 210 phosphatidylethanolamines (PE), 125 phosphatidylserines (PS), and 125 diglycerides (DG) (Figure 1B). The information of all identified lipid molecules was shown in Table S1. The principal component analysis (PCA) plot clearly shows distinct clusters of the four treatment groups (CG, Cold *C. gigas* ; CA, Cold *C. angulata* ; HG, Heat *C. gigas* ; HA, Heat *C. angulata* ) (Figure 1C). Compared to acute cold stress, there were 496 significantly upregulated lipid molecules and 125 downregulated lipid molecules after heat stress in *C. gigas* , while in *C. angulata* , there were 320 upregulated lipid molecules and 154 downregulated lipid molecules (Figure 1D,E). To better screen differential lipid substances, we conducted Orthogonal Partial Least Squares Discriminant Analysis (OPLS‐DA) to establish a model depicting the relationship between lipid expression levels and sample categories (Figure S2). In this experiment, a VIP value > 1 and a *p* < 0.05 were used as the criteria for significant differential lipid molecule selection in OPLS‐DA. Information regarding the significant differential lipid molecules can be found in Table S2. The results indicated that among the PE, PC, Cer, and TG lipid classes, there were the highest numbers of differential lipid molecules between the heat and cold stress responses in *C. gigas* and *C. angulata* (Figure 1F,G). In terms of percentage composition, after cold stimulation, sphingosine (SPH) increased from 20.457% to 25.575% in *C. gigas* and from 22.821% to 23.788% in *C. angulata* , becoming the substance with the highest content in cell membranes (Figure S3). These results indicated that there were differences in the overall composition of plasma membrane lipids between *C. gigas* and *C. angulata* , and that they were influenced by short‐term acute temperature stress.

### 3.2 The Lipid Content of Plasma Membranes in *C. gigas* and *C. angulata* Responded to Acute Temperature Stress

**FIGURE 2:**
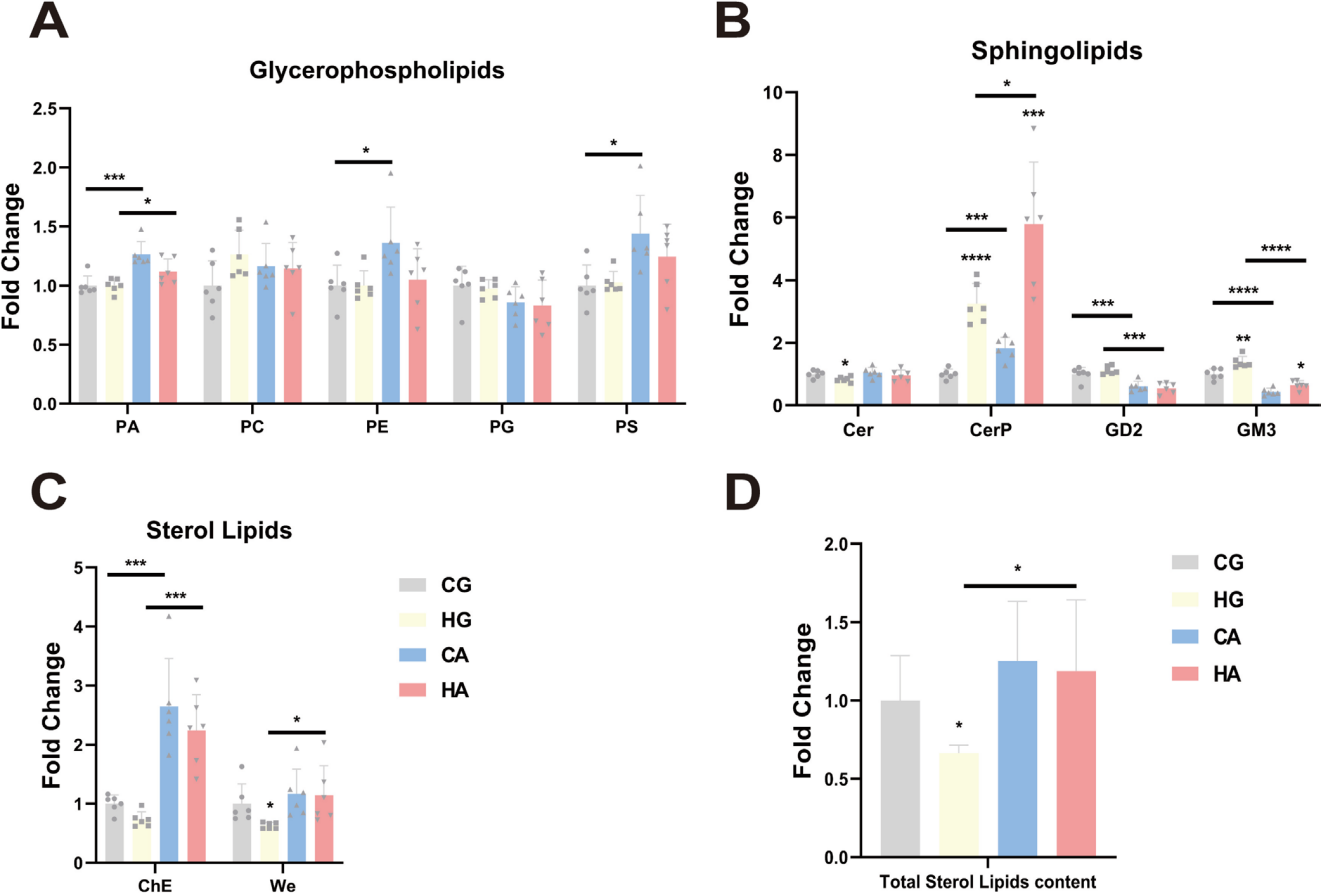
Short‐term acute temperature stress altered the content of lipid subclasses in plasma membrane. (A–D) The fold change of lipid subclasses. Gray bars represented cold‐stressed *Cangulata gigas* , light yellow bars represented heat‐stressed *C. gigas* , light blue bars represented cold‐stressed *Cangulata angulata* , and red bars represented heat‐stressed *C. angulata* . (A) glycerophospholipids, (B) sphingolipids, (C) sterol lipids, (D) total sterol lipids content. The error bars represented SE. Significant differences among groups were marked with *p < 0.05, **p < 0.01, ***p < 0.001, ****p < 0.0001.

The acute temperature changed the relative content of various lipid classes in two closely related oyster species. We divided lipids into three main categories: glycerophospholipids, sphingolipids, and sterol lipids, and the relative content of all subcategories was displayed in Table S3 and Figure S4. The lipid subclasses of glycerophospholipids in the two oyster species did not show significant changes under ambient temperature variations. However, between the two oyster species, we found that the content of phosphatidic acid (PA), PE, and PS in *C. angulata* was significantly higher than in *C. gigas* , while PC and phosphatidylglycerol (PG) showed no significant differences (Figure 2A). Changes in the content of sphingolipids were more pronounced both under temperature stress and in interspecies comparisons (Figure 2B). After heat stress, the levels of Cer significantly decreased in *C. gigas* , with a decreasing trend also observed in *C. angulata* , but the interspecies difference was not significant. The content of ceramide phosphates (CerP) significantly increased in response to heat stress compared to cold stress in both two oyster species, with *C. angulata* consistently showing significantly higher levels than *C. gigas* (Figure 2B). We observed significant interspecies differences in cholesterol esterase (ChE) content, with *C. angulata* showing significantly higher content than *C. gigas* , which responded with an increase in heat stress (Figure 2C). In addition to assessing the content of lipid subclasses, we also compared the content of total sterol lipids, which were related to membrane fluidity (Figure 2D). After heat stress, both *C. gigas* and *C. angulata* showed a decrease in the content of total sterol lipids, with a significant decrease observed in *C. gigas* . Although higher content was observed in the relatively heat tolerant *C. angulata* , significance was only observed in the HG vs. HA group. These findings suggested that acute temperature stress induced considerable alterations in the content of lipid species and that there were differences between the two oyster species.

### 3.3 The Relatively Cold Tolerant *C. gigas* Preferred to Change the Length of Fatty‐Acyl Chains of Glycerophospholipids to Adapt Acute Temperature

**FIGURE 3:**
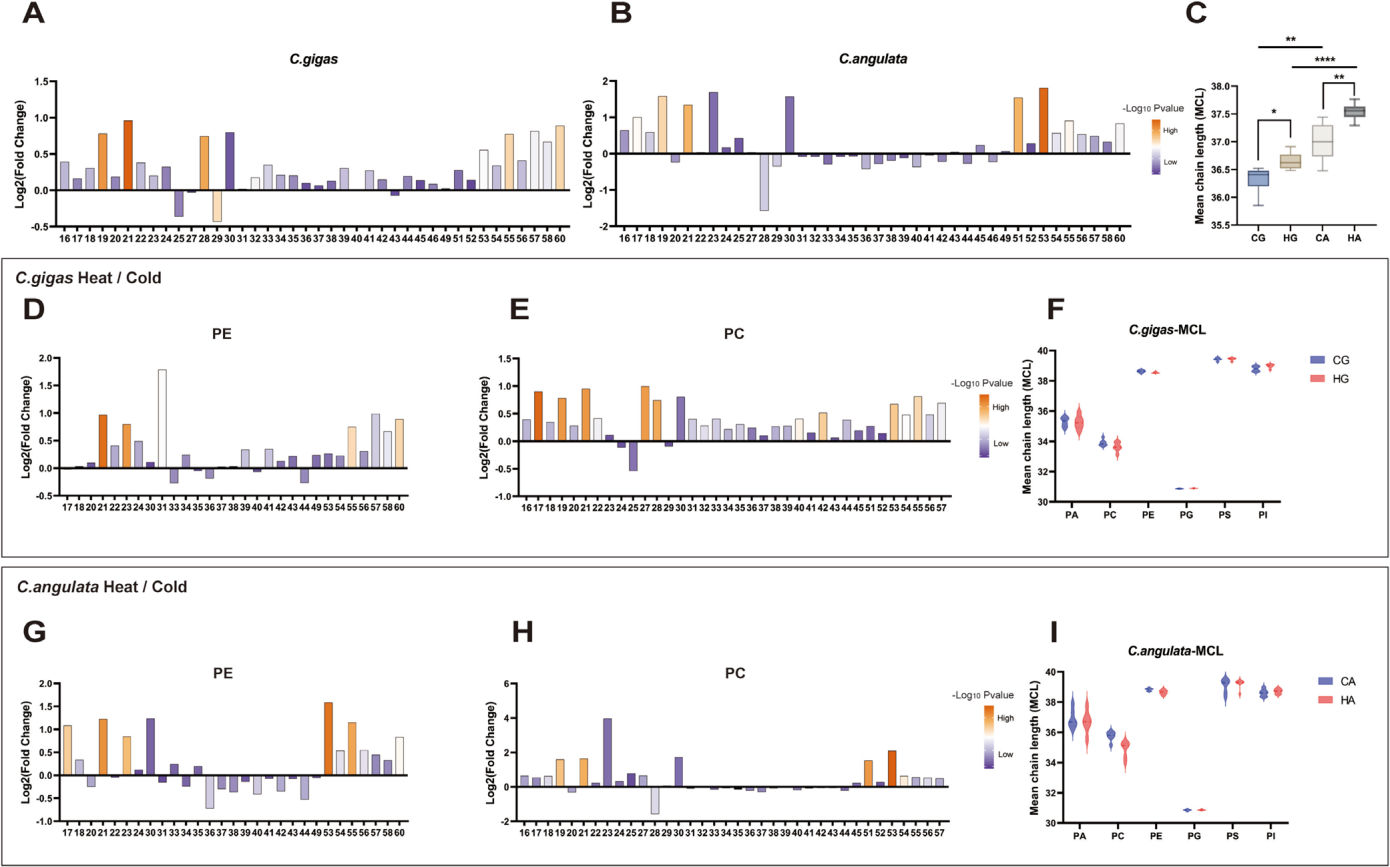
Short‐term acute temperature stress mediated changes in length of fatty‐acyl chains of glycerophospholipids in *Cangulata gigas* and *Cangulata angulata* . (A, B) The fold change of total glycerophospholipids content with different carbon atom numbers between heat stress versus cold stress treated in *C. gigas* (A) and *C. angulata* (B). The *x*‐axis represented lipid molecules with different carbon atom numbers, and the *y*‐axis represented the Log_2_fold change after heat stress. Color variations indicated the magnitude of −log_10_(*p* value). (C) The Mean chain length (MCL) of total glycerophospholipids. (D, E, G, H) The fold change of glycerophospholipid subclasses (PE, PC) content with different carbon atom numbers in *C. gigas* and *C. angulata* . (F, I) The MCL of glycerophospholipid subclasses in *C. gigas* and *C. angulata* . Blue bars represented short‐term cold stress treatment, while red bars represented heat stress treatment. The error bars represented SE. Significant differences among groups were marked with *p < 0.05, **p < 0.01, ***p < 0.001, ****p < 0.0001.

Glycerophospholipids, key and major constituents of cell membranes, play a critical role in modulating membrane fluidity and equilibrium (Ernst et al. 2016; Hishikawa 2014). Therefore, subsequent analysis focused on the changes in chain length and unsaturation of glycerophospholipids, which were important factors influencing membrane fluidity to adapt temperature variations (Winnikoff et al. 2021). We found that lipid molecules with different chain lengths of glycerophospholipids generally increased in content compared to cold stress after heat stress in *C. gigas* , except for lipid molecules with carbon atom numbers of 25, 27, 29, and 43 (Figure 3A). However, in *C. angulata* , lipid molecules with carbon atom numbers ranging from 28 to 49 mostly decreased, except for 28 which showed a minimal decrease (Figure 3B). The mean chain length (MCL) of total glycerophospholipids significantly increased in two oyster species after heat stress, with *C. angulata* significantly higher than *C. gigas* both in heat stress and cold stress treatment groups (Figure 3C). Further focusing on the changes in chain length of glycerophospholipid subclasses, it was observed that the chain length of the majority of carbon atoms in lipid molecules exhibited an increasing trend consistent with the MCL of total glycerophospholipids (Figure 3D–I). From the most abundant PE and PC, it was found that the chain length increase was more pronounced in *C. gigas* compared to *C. angulata* (Figure 3D,E,G,H), while lower abundance components exhibited different trends (Figure S5). The lipid molecules with carbon atom numbers of 21, 23, and 55 in PE significantly increased after heat stress in both *C. gigas* and *C. angulata* . Numbers ranging from 33 to 49 showed a decreasing trend in *C. angulata* and a less significant increase in *C. gigas* . Similarly, in PC, lipid molecules with carbon atom numbers of 19, 21, and 53 significantly increased after heat stress, while numbers ranging from 31 to 45 showed a decreasing trend in *C. angulata* . Two oyster species tended to prefer altering lipid molecules with relatively low and high carbon atom numbers, while lipid molecules around 30 to 50 exhibited minor changes, which even showed a declining trend in response to heat stress in *C. angulata* . The MCL of various subclasses of glycerophospholipids did not undergo significant changes in response to acute temperature, which indicated that the change in MCL of total glycerophospholipids was not solely attributed to a specific subclass (Figure 3F,I). These results indicated that under acute temperature stress, two oyster species exhibited varying degrees of changes in glycerophospholipids chain lengths to alter membrane fluidity, suggesting different strategies in lipid molecule chain length alterations.

### 3.4 The Relatively Heat Tolerant *C. angulata* Preferred to Change the Fatty Acid Unsaturation of Glycerophospholipids to Adapt Acute Temperature

**FIGURE 4:**
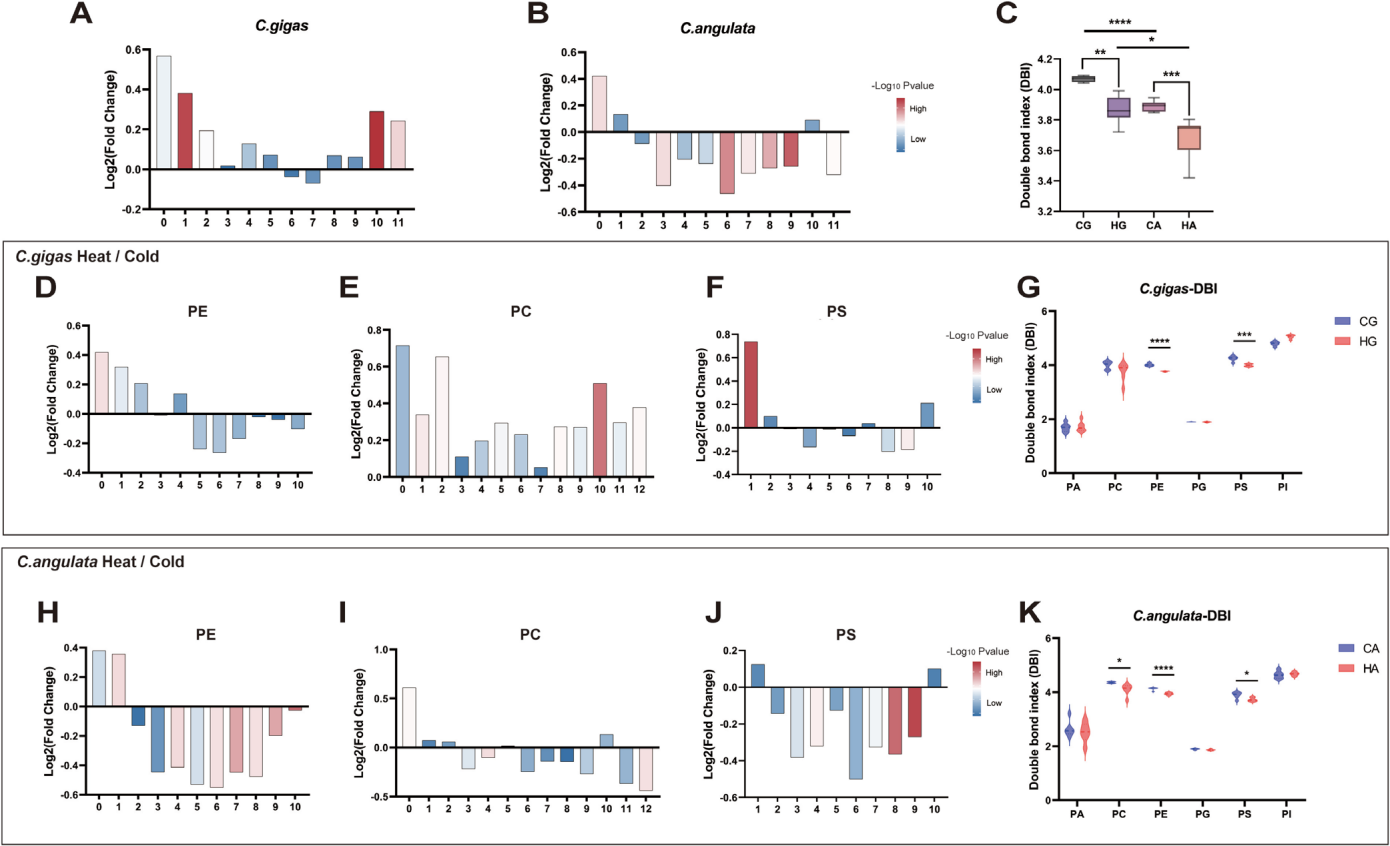
Short‐term acute temperature stress mediated changes in the fatty acid unsaturation of glycerophospholipids in *Cangulata gigas* and *C. angulata* . (A, B) The fold change of total glycerophospholipids content with different numbers of double bonds between heat stress versus cold stress treated in *C. gigas* (A) and *C. angulata* (B). The *x*‐axis represented lipid molecules with different numbers of double bonds, and the *y*‐axis represented the log_2_ fold change after heat stress. Color variations indicated the magnitude of −log_10_(*p* value). (C) The Double bond index (DBI) of total glycerophospholipids. (D–F, H–J) The fold change of glycerophospholipid subclasses (PE, PC, PS) content with different numbers of double bonds in *C. gigas* and *C. angulata* . (G, K) The DBI of glycerophospholipid subclasses in *C. gigas* and *C. angulata* . Blue bars represented short‐term cold stress treatment, while red bars represented heat stress treatment. The error bars represented SE. Significant differences among groups were marked with *p < 0.05, **p < 0.01, ***p < 0.001, ****p < 0.0001.

Additionally, we evaluated the unsaturation of glycerophospholipids, which was a recognized important factor influencing membrane fluidity (Ernst et al. 2016). The results showed that in *C. angulata* , the saturated glycerophospholipids content increased, while lipids with different numbers of double bonds showed a decreasing trend, except for those with 1 and 10 double bonds, which showed a slight increase after heat stress (Figure 4B). In *C. gigas* , saturated and unsaturated glycerophospholipids both showed an increasing trend compared to cold stress after heat stress, except for lipids with 6 and 7 double bonds (Figure 4A). Although the number of double bonds increased in *C. gigas* after heat stress based on the content changes, the saturated fatty acid content showed the highest increase in multiples. Therefore, the Double Bond Index (DBI) significantly decreased after heat stress in both *C. gigas* and *C. angulata* (Figure 4C). We also observed that the DBI in *C. gigas* was significantly higher than in *C. angulata* under both heat and cold stress conditions. Focusing on the unsaturation changes in glycerophospholipid subclasses, we observed that except for PC and Phosphatidylinositol (PI) in *C. gigas* , the unsaturation levels in the other subclasses decreased in response to heat stress (Figure 4D–K, Figure S6). Among the top three subclasses of glycerophospholipids with the highest content (PE, PC, and PS), it can be observed that saturated lipids (0) and lipids with low double bond numbers (1, 2) showed an increasing trend after heat stress. However, lipids with high double bond numbers (3–12) exhibited a significant decrease in two oyster species, except for PC in *C. gigas* (Figure 4D–FH–J). The DBI of glycerophospholipid subclasses was also observed to exhibit a decreasing trend after heat stress, which indicated that the unsaturation level changes in glycerophospholipids respond to acute temperature. Specifically, significant decreases were observed in PE and PS in *C. gigas* , and in PC, PE, and PS in *C. angulata* (Figure 4G,K). These results suggested that glycerophospholipids exhibit different adaptive strategies in response to acute temperature stress, similar to chain length variations, in *C. gigas* and *C. angulata* .

### 3.5 Identification of Key Lipid Molecules in Plasma Membrane for Short‐Term Cold and Heat Adaptation

**FIGURE 5:**
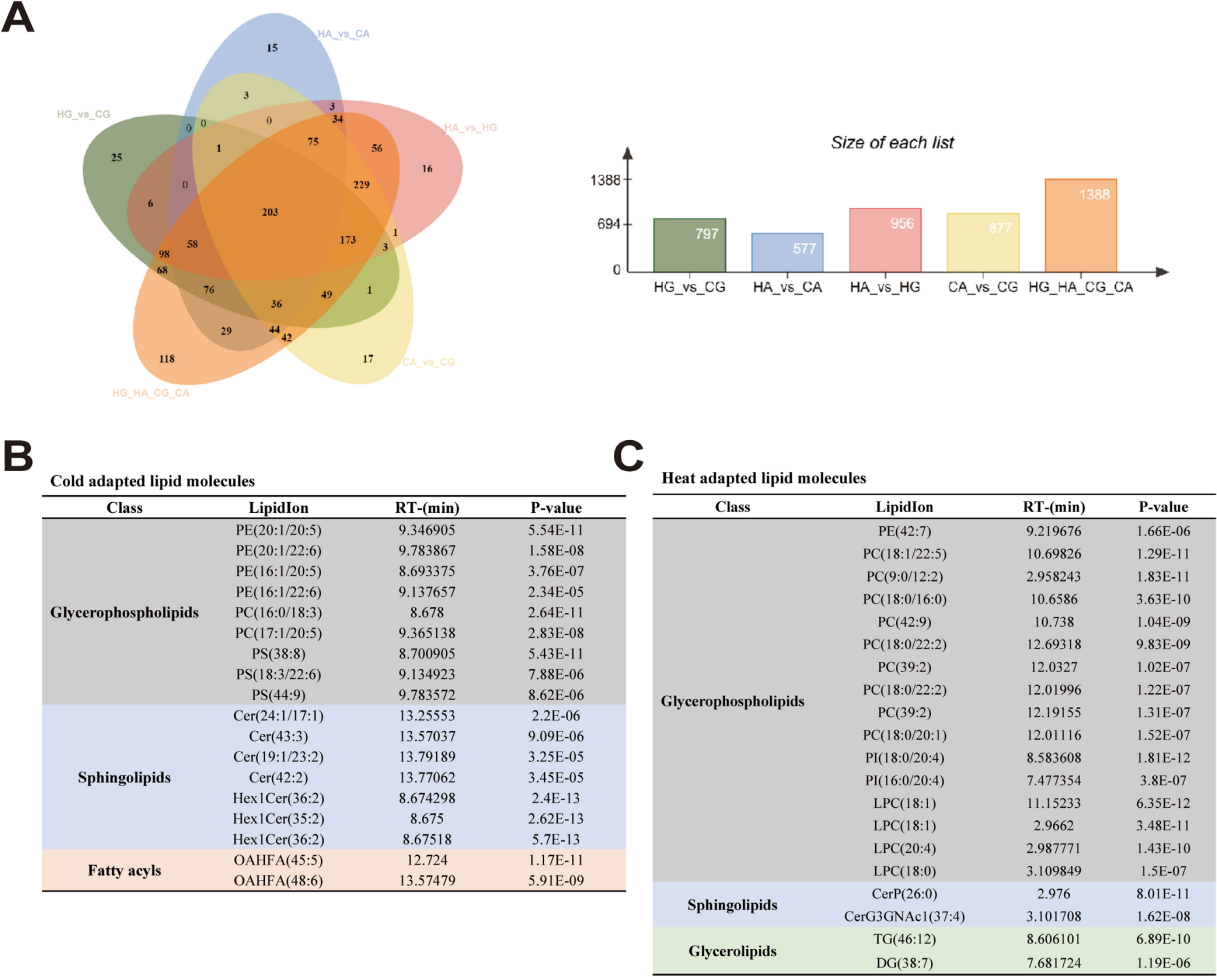
The identification of key short‐term cold and heat adapted lipid molecules in plasma membrane lipid. (A) The intersecting key lipid molecules among different treatment groups selected by OPLS‐DA model. (B, C) The information of key cold and heat adapted lipid molecules identified by interspecific differences driven by habitat temperature variations and the environmental shifts caused by acute temperature stress.

The key lipid molecules crucial for short‐term temperature adaptation were identified through the selection of significantly differential lipid species in the OPLS‐DA of four comparative groups (HG/CG, HA/CA, HA/HG, CA/CG, Table S2). The intersection of key lipid molecules identified in different treatment groups was shown in Figure 5A. We considered lipid molecules that showed increased levels after heat stress in two oyster species (HG>CG, HA>CA) and were present in higher content in the relatively heat tolerant *C. angulata* compared to *C. gigas* (HA>HG, CA>CG) as heat adapted lipid molecules, while conversely as cold adapted lipid molecules (Table S4). Cold adapted lipid molecules were mainly composed of glycerophospholipids and sphingolipids, including 4 PE, 3 PS, 2 PC, 4 Cer, and 3 Hex1Cer (Figure 5B). Heat adapted lipid molecules are primarily composed of glycerophospholipids, including 9 PC, 4 LPC, 2 PI, and 1 PE (Figure 5C). We found that compared to heat adapted lipid molecules, the glycerophospholipid fatty acids of cold adapted lipid molecules had a higher number of double bonds, which may help adapt to cold stress. In heat adapted lipid molecules, PC was the main component, followed by LPC, which was a breakdown product of PC. Therefore, PC may play a key role in short‐term heat adaptation.

## 4 Discussion

In the context of frequent extreme marine climate events, the survival challenges posed to organisms by acute temperature stress have received significant attention (Harris et al. 2018). Indoor acute temperature stress simulations, effectively isolating environmental interference while accurately replicating extreme marine temperature variations, have been widely applied in ecological studies, as evidenced by research on organisms like *Euphausia superba* (Huenerlage et al. 2016), mussel (Sorte et al. 2019) and corals (Sweet et al. 2021). We selected the plasma membrane, which may be the first cellular structure to detect change in ambient temperature (Ernst et al. 2020), as our target to explore temperature adaptation strategies in marine organisms by characterizing membrane lipid remodeling under acute temperature stress in two related oyster species, *C. gigas* and *C. angulata* , with divergent temperature tolerance. Previous comparative analyses revealed that the relatively cold tolerant *C. gigas* exhibited significantly higher levels of crude lipid content, free fatty acids content, and C18:1 oleic acid desaturation index compared to *C. angulata* (Li et al. 2021; Wang et al. 2023; Wang et al. 2021). Concurrently, lipid catabolism genes were preferentially upregulated in *C. angulata* , whereas lipid anabolism genes dominated in *C. gigas* (Li et al. 2021). These findings demonstrate that lipid traits play an important role in temperature adaptation, while the changes in membrane fluidity mediated by fine‐scale lipid remodeling are more closely related to temperature adaptation. The temperature‐responsive remodeling of membrane lipids, known as homeoviscous adaptation, was first discovered in bacteria (Sinensky 1974) and found to support the survival of poikilothermic organisms, such as nematodes and flies, under low temperature conditions (Goto and Katagiri 2011; Guschina and Harwood 2006; Hayward et al. 2007). The fluidity regulation via membrane lipid remodeling involved intricate mechanisms; here, we dissected this process by integrating lipid subclass content, acyl chain length, and unsaturation levels of glycerophospholipids.

Short‐term acute temperature stress did not produce a significant consistent trend in the changes of membrane lipid subclasses content between the two oyster species. As the most important components of the cell membrane, glycerophospholipids showed low sensitivity to short‐term temperature stress but exhibited more pronounced differences between the two oyster species. The elevated levels of PA, PE, and PS in *C. angulata* compared to *C. gigas* reflected species‐specific membrane adaptations shaped by evolutionary pressures. The smaller head group and partial positive charge of PE enabled tighter packing with acyl chains, and its higher content increased membrane rigidity, thereby aiding *C. angulata* in adapting to high temperature environments (Dawaliby et al. 2016; Renne and de Kroon 2018). However, the lack of acute temperature responsiveness in glycerophospholipid content implied that rapid adaptation mechanisms may prioritize dynamic lipid modifications over wholesale changes in glycerophospholipid abundance. Compared to glycerophospholipids, sphingolipid content exhibits significant interspecific variation between the two oyster species and demonstrates acute temperature responsiveness, reflecting its critical role in membrane lipid remodeling‐mediated temperature adaptation (Zhu et al. 2023). The significant increase in the precursor of the important signaling molecule sphingosine‐1‐phosphate S1P (SPH) proportion after cold stress, becoming the most abundant lipid, suggested its role in activating key lipid pathways like the AdipoR‐Srebp/Ppar axes, crucial for membrane fluidity and temperature adaptation (Hsing et al. 2024; Ruiz et al. 2022). The high content of CerP in relatively heat tolerant *C. angulata* , along with its significant increase in both oyster species after heat stress, suggested that CerP may play a critical role as a membrane sphingolipid in high temperature adaptation. High content of sphingolipids, due to their elevated phase transition temperature, was shown to increase membrane rigidity, as demonstrated in bacterial and plant studies (Fabri et al. 2020; Xu et al. 2023). Additionally, as a key regulator of membrane fluidity, sterol lipids Che did not show significant changes under temperature stress, while they were consistently higher in *C. angulata* compared to *C. gigas* . Sterol lipids, which garnered significant attention due to their unique chemical structure that restricted the disordered motion of phospholipids and reduced membrane fluidity, played a critical role in temperature adaptation, as evidenced in aquatic animals such as shrimp (Sánchez et al. 2001), steelhead trout (Wijekoon et al. 2021) and blue mussel (Fokina et al. 2018).

In response to acute temperature stress, the two oyster species tended to divergent lipid modification approaches of membrane glycerophospholipids to regulate membrane fluidity, highlighting their unique adaptation strategies. The relatively cold tolerant *C. gigas* favored altering glycerophospholipid acyl chain length, whereas *C. angulata* preferentially modified glycerophospholipid fatty acid unsaturation. Long‐chain fatty acids promoted tighter glycerophospholipid packing through stronger hydrophobic interactions, thereby reducing membrane fluidity for high‐temperature adaptation, while shorter chains were utilized to enhance fluidity under cold stress, which was proved in bacteria and yeast (Kaur et al. 2015; Suutari et al. 1997; Trenti et al. 2022). Our results also revealed an increase in membrane glycerophospholipid acyl chain length at both the overall and subclass levels under high‐temperature stress in the two oyster species, which contributed to reduced membrane fluidity and maintained normal functionality (Wu et al. 2023; Zhang et al. 2024). Furthermore, the chain length of membrane glycerophospholipids in the relatively warm‐adapted *C. angulata* was significantly higher than that in *C. gigas* , which may suggest that membrane lipid chain length was an important temperature adaptive trait that can help oysters cope with changes in membrane fluidity induced by environmental temperature variations during long‐term adaptation processes (Ghaffari et al. 2019; Li et al. 2021). Changes in fatty acid chain length may exhibit species‐specific preferences, with medium‐chain lipid molecules (28–49 carbons) showing limited responsiveness to acute temperature changes. After heat stress, both *C. gigas* and *C. angulata* exhibited significant increases in lipid molecules with carbon numbers of 21, 23, and 55 in PE, and 19, 21, and 53 in PC, further demonstrating the preference for modifying acyl chain lengths. However, the molecular regulatory mechanisms underlying the preferential modification of membrane lipid acyl chain lengths remain to be elucidated. Additionally, the DBI of total membrane glycerophospholipids, a key factor influencing membrane fluidity, showed a significant decrease at the overall level in both oyster species after heat stress. Double bonds reduced membrane fluidity by restricting the rotational freedom of fatty acid chains and facilitating tighter lipid packing to adapt to temperature change, which was widely validated in diverse organisms, including fish (Black et al. 2025; Gonzalez et al. 2013), plants (Singh et al. 2022; Yin et al. 2024), and model species (Ballweg et al. 2020; Bussmann et al. 2023; Harayama and Antonny 2023). Notably, at the phospholipid subclass level, *C. gigas* exhibited an increase in both saturated and unsaturated lipid molecules after heat stress, whereas the overall DBI decreased, with *C. angulata* showing a more pronounced decline. Comparative analysis between the two oyster species demonstrated significantly higher membrane lipid unsaturation in *C. gigas* compared to *C. angulata* under cold and heat stress, which, together with acyl chain length modifications, regulated membrane fluidity during temperature adaptation. Therefore, both allopatric congeneric oyster species conformed to the principles of homeoviscous adaptation by modulating acyl chain length and unsaturation under acute temperature stress, but they diverged in preferred strategies for lipid molecular remodeling. However, the evolutionary drivers and molecular mechanisms underlying adaptation strategic choices in *C. gigas* and *C. angulata* remain to be further investigated.

After screening for significantly different lipid molecules using the OPLS‐DA model, we identified key lipids associated with heat and cold acute temperature adaptation. The key lipid molecules screened based on two factors—interspecific differences driven by habitat temperature variations and the environmental shifts caused by acute temperature stress—were more credible. Cold‐adapted lipid molecules, especially glycerophospholipids, had significantly more double bonds than heat‐adapted lipid molecules, while chain length variations were minimal, indicating that double bonds may contribute more significantly to temperature adaptation in oysters (Chen and Ho 1985; Niemelä et al. 2006; Wang et al. 2023). Additionally, cold‐adapted lipid molecules were primarily composed of glycerophospholipids and sphingolipids, whereas heat‐adapted lipid molecules were dominated by glycerophospholipids, particularly PC and LPC, further indicating the critical role of lipid composition in regulating membrane fluidity and temperature adaptation (Bennett et al. 2018; Sinensky 1974; Wu et al. 2023). The identified heat and cold‐adapted lipid molecules can serve as key biomarkers for assessing membrane fluidity dynamics and temperature adaptation potential in marine organisms under global warming. However, this study lacks comprehensive analysis of the regulatory network controlling membrane fluidity and corresponding experimental validation. Our previous research has demonstrated that stearoyl‐CoA desaturase (SCD) and elongation of very long‐chain fatty acids (ELOVL) enzymes regulate fatty acid unsaturation and chain length in oysters, respectively (Liu et al. 2023; Wang et al. 2023). These findings suggest SCD and ELOVL may serve as promising research targets for future investigations.

## 5 Conclusion

**FIGURE 6:**
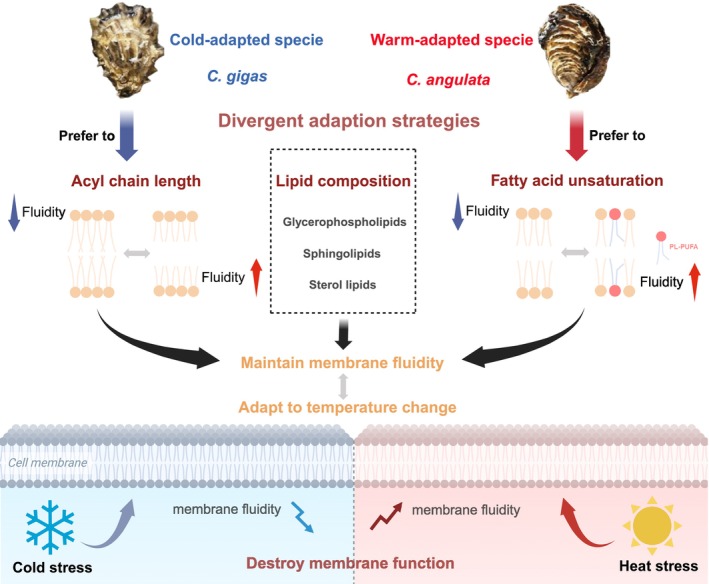
Schematic representation of divergent adaptation strategies of membrane lipid remodeling for acute temperature adaptation in *Cangulata gigas* and *Cangulata angulata* . Heat and cold stress increased and decreased membrane fluidity, respectively, thereby disrupting normal membrane biological functions. As poikilothermic organisms, oysters responded to environmental stress by remodeling lipid composition, acyl chain length and unsaturation—a process known as homeoviscous adaptation. The cold‐adapted species *C. gigas* preferentially modulated acyl chain length, whereas the warm‐adapted species *C. angulata* favored altering unsaturation to maintain membrane fluidity. The two oyster species maintained membrane fluidity in response to acute temperature changes by favoring divergent adaptation strategies.

In conclusion, we characterized the changes in plasma membrane lipids of two related oyster species *C. gigas* and *C. angulata* under acute temperature stress through lipidomics for the first time. Our results revealed varying degrees of changes in glycerophospholipids, sphingolipids, and sterol lipids under acute temperature stress. Although glycerophospholipids were the primary membrane components, sphingolipids and sterol lipids may play more significant roles in short‐term temperature adaptation. The changes in acyl chain length and unsaturation of glycerophospholipids in *C. gigas* and *C. angulata* conformed to the principles of homeoviscous adaptation. However, the two oyster species exhibited divergent preferences in lipid remodeling under acute temperature stress: northern oyster *C. gigas* preferred to alter chain length, whereas southern oysters *C. angulata* preferred to modify double bond numbers (Figure 6). The revelation of different membrane lipid adaptation strategies deepened the understanding of the role of plasma membrane fluidity in temperature adaptation among marine organisms.

## Conflicts of Interest

The authors declare no conflicts of interest.

## Supporting information


**Data S1:** Supplementary Figures S1–S6.

**Table S1:** Information of the identified lipid molecule.

**Table S2:** Information of significantly different lipid molecules.

**Table S3:** The relative content of all subcategories in four group.

**Table S4:** Information of key heat and cold adapted lipid molecules.

## Data Availability

There is no sequencing raw data for this study.
