# Supplementary material for: Membrane Lipid Remodeling Strategies Regulate Fluidity for Acute Temperature Adaptation in Oysters
Source: Evol Appl. 2025 Sep 13;18(9):e70156. doi: 10.1111/eva.70156 (PMC12432411; doi:10.1111/eva.70156)
Supplement: Supplementary file 1 — Data S1: Supplementary Figures S1–S6. [file EVA-18-e70156-s002.docx]

**Supplemental File**

Supplementary figures


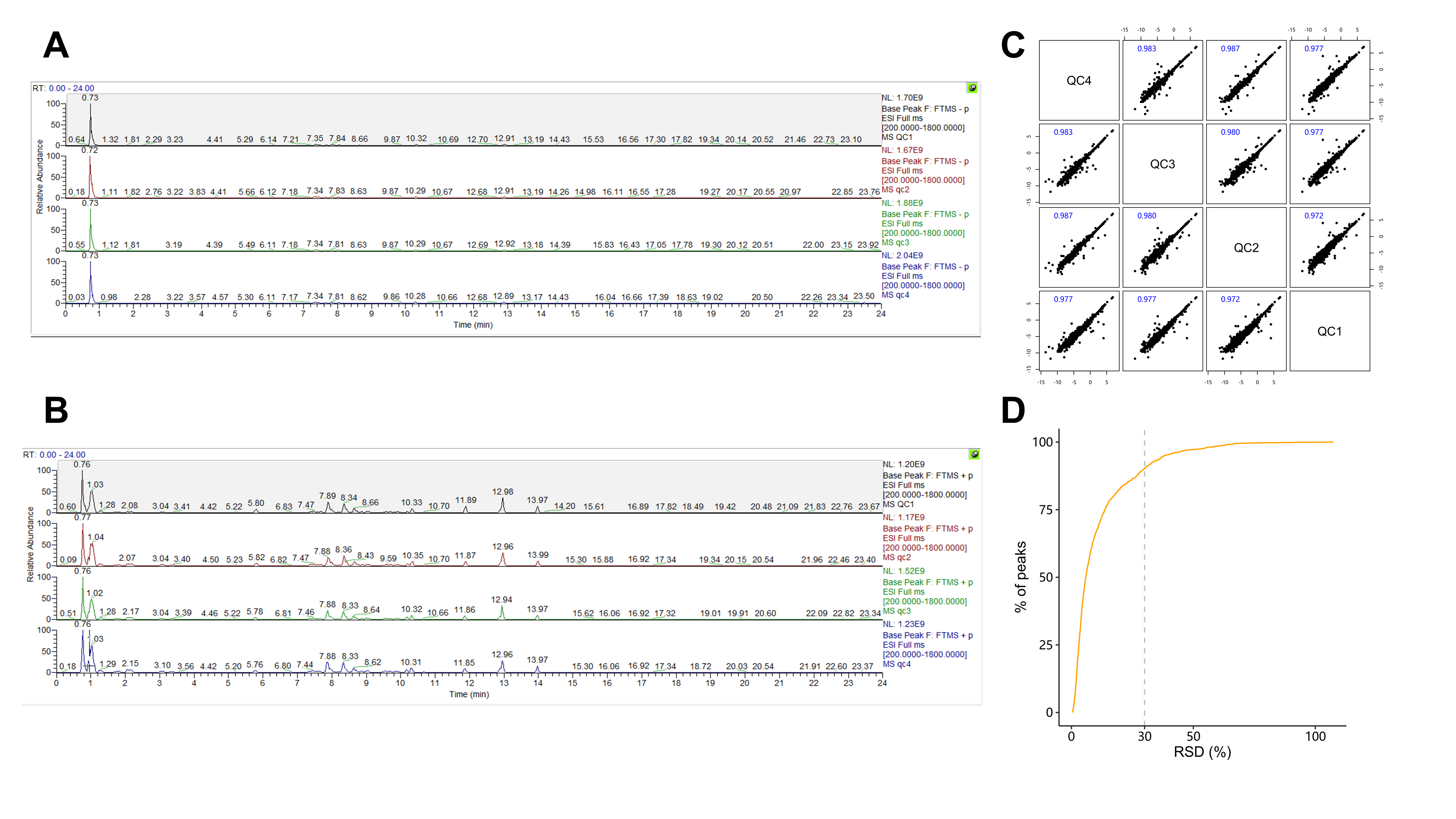


Supplementary Fig. S1 Quality control assessment of the lipidomic dataset. (A) Overlap of base peak chromatograms (BPC) in positive ion mode. (B) Overlap of base peak chromatograms (BPC) in negative ion mode. The experimental results indicate that the chromatographic peak intensities and retention times of each QC sample largely overlap, suggesting good experimental reproducibility. (C) Correlation plot of QC samples. Each point in every cell represents an ion peak (metabolite) extracted from the QC sample, where the x-axis and y-axis represent the logarithmic values of the ion peak signal intensities. The correlation coefficients between QC samples are all above 0.9. (D) Relative standard deviations of the QC samples. The proportion of peaks in the QC samples with RSD ≤ 30% to the total number of peaks in the QC samples is above 80%, indicating good stability of the instrument analysis system.


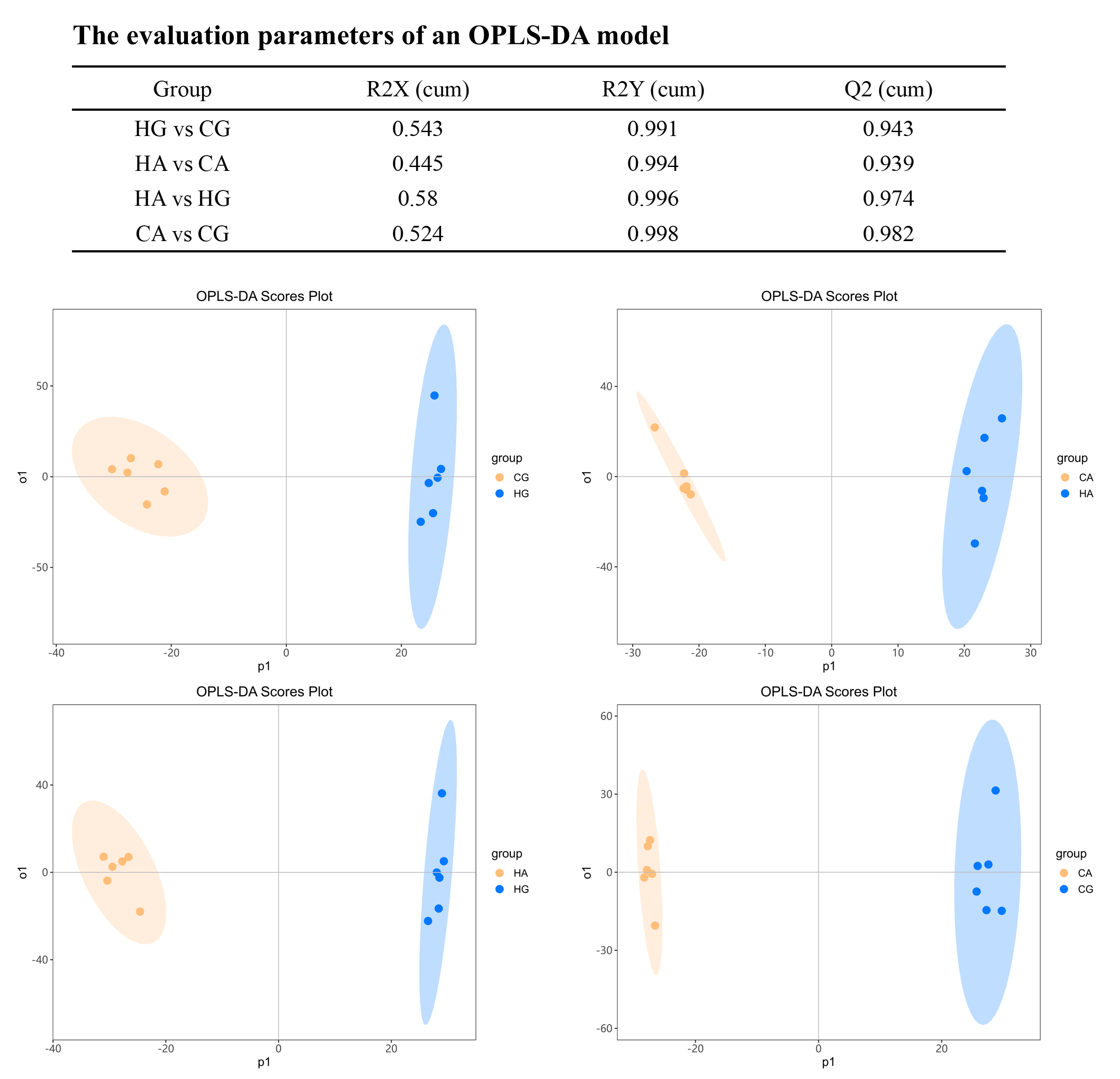


Supplementary Fig. S2 The evaluation parameters and score Plot of OPLS-DA Model.


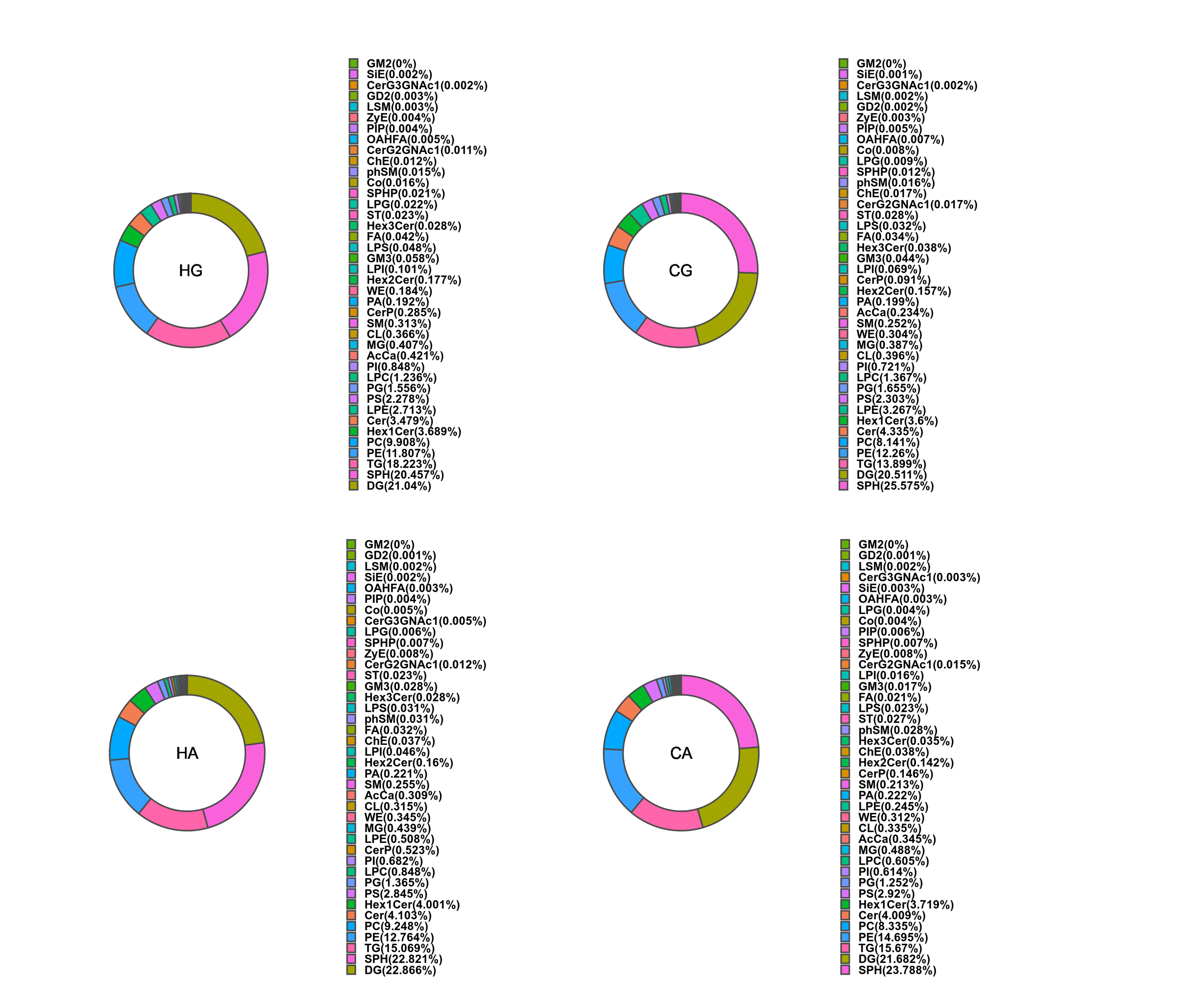


Supplementary Fig. S3 The lipid composition of each treatment group.


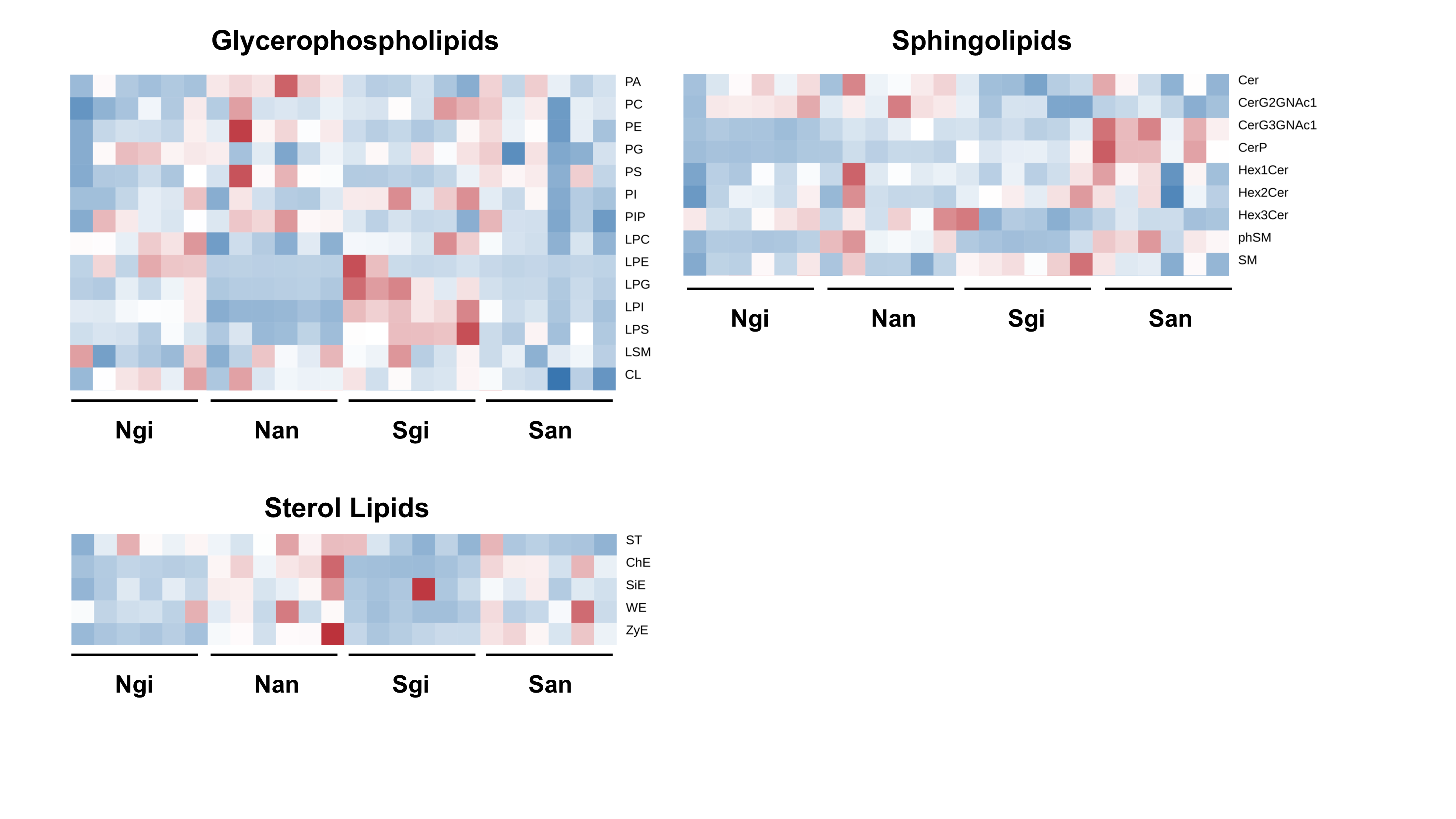


Supplementary Fig. S4 The lipid composition of each treatment group.


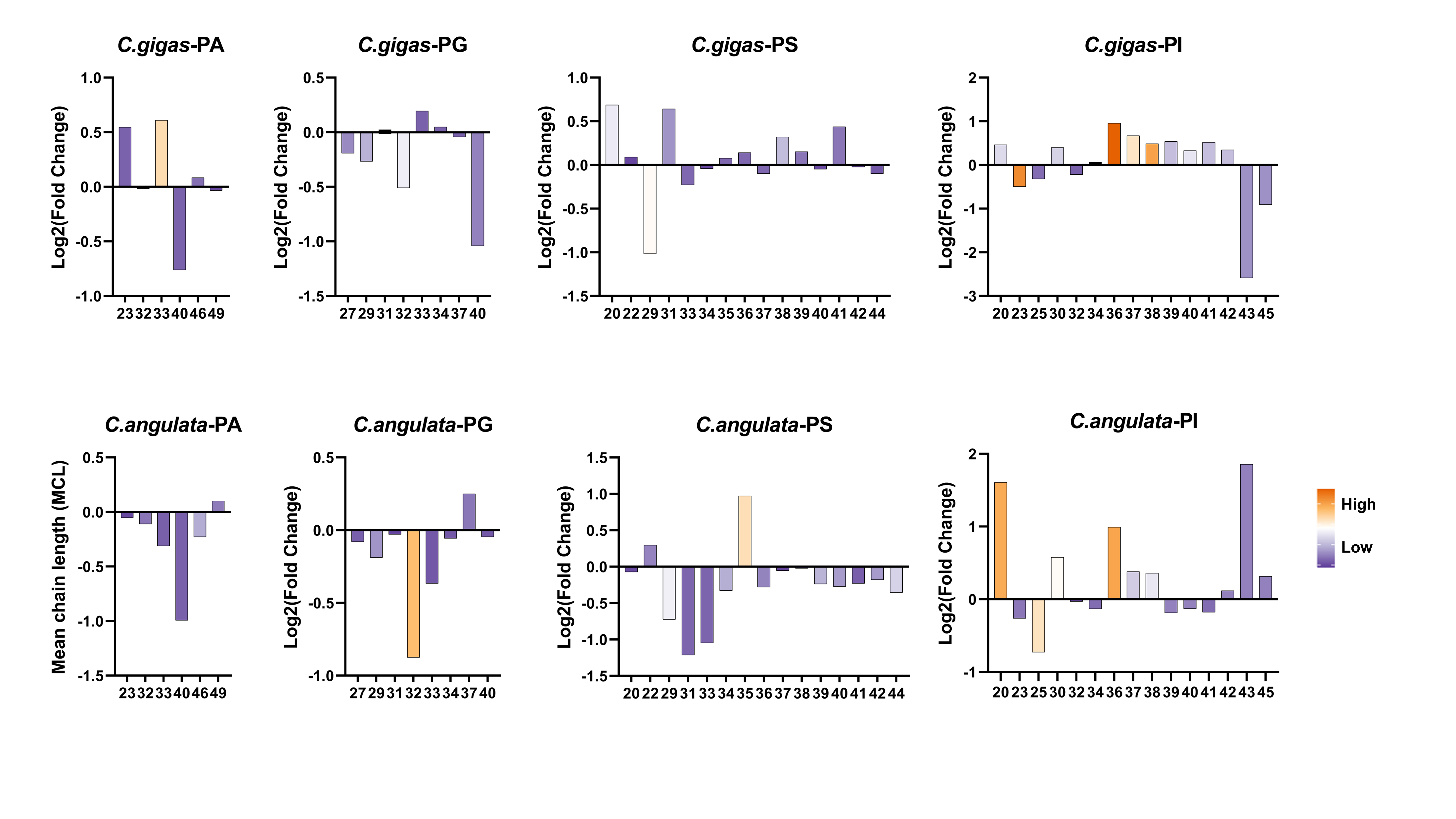


Supplementary Fig. S5 The fold change of glycerophospholipid subclasses content with different carbon atom numbers between heat stress versus cold stress treated in *C. gigas* and *C. angulata*. The x-axis represented lipid molecules with different carbon atom numbers, and the y-axis represented the Log2 fold change after heat stress. Color variations indicated the magnitude of -log_10_ (*P* value).


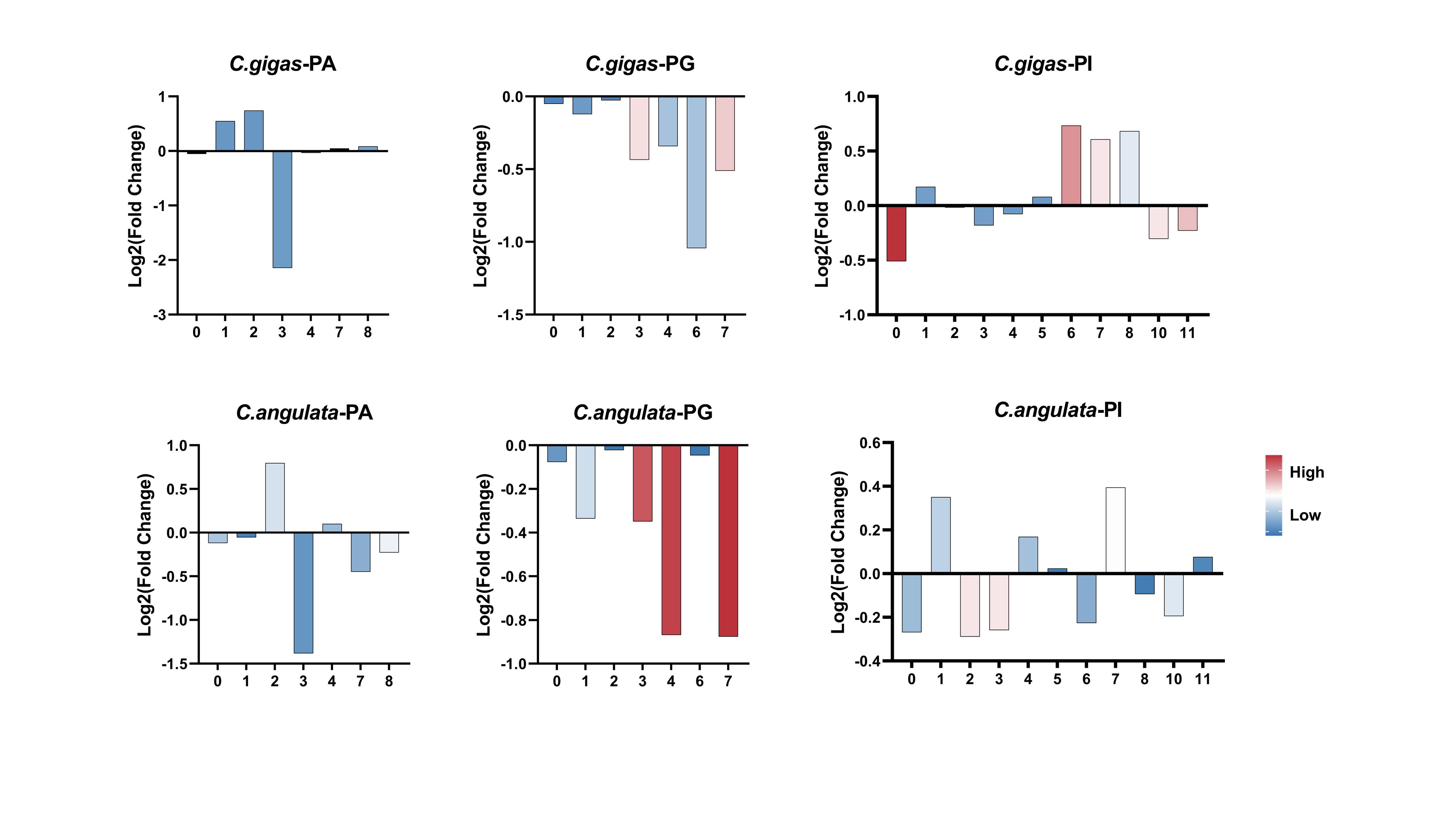


Supplementary Fig. S6 The fold change of glycerophospholipid subclasses content with different numbers of double bonds between heat stress versus cold stress treated in *C. gigas* and *C. angulata*. The x-axis represented lipid molecules with different numbers of double bonds, and the y-axis represented the Log_2_ fold change after heat stress. Color variations indicated the magnitude of -log_10_(*P* value).
